# Urinary Proteome of Newborn Calves—New Potential in Non-Invasive Neonatal Diagnostic

**DOI:** 10.3390/ani10081257

**Published:** 2020-07-24

**Authors:** Alicja Dratwa-Chałupnik, Katarzyna Wojdyła, Małgorzata Ożgo, Adam Lepczyński, Katarzyna Michałek, Agnieszka Herosimczyk, Adelina Rogowska-Wrzesińska

**Affiliations:** 1 Department of Physiology, Cytobiology and Proteomics, Faculty of Biotechnology and Animal Husbandry, West Pomeranian University of Technology Szczecin, Klemensa Janickiego 29, 71-270 Szczecin, Poland; malgorzata.ozgo@zut.edu.pl (M.O.); adam.lepczynski@zut.edu.pl (A.L.); katarzyna.michalek@zut.edu.pl (K.M.); agnieszka.herosimczyk@zut.edu.pl (A.H.); 2 Department of Biochemistry and Molecular Biology, University of Southern Denmark, Campusvej 55, M-DK-5230 Odense, Denmark; wojdyla.kat@gmail.com (K.W.); adelinar@bmb.sdu.dk (A.R.-W.)

**Keywords:** two-dimensional electrophoresis, MALDI-TOF-TOF-MS/MS, one-dimensional electrophoresis, LC-MS/MS, urine proteins

## Abstract

**Simple Summary:**

Urine is an underappreciated biological sample in the context of health monitoring of newborn calves. It can be collected noninvasively and used for diagnostic purposes, which is an attractive option in veterinary examination. The establishment of urinary protein maps of healthy newborn calves is important for diagnosing and monitoring the progression of various diseases. The aim of this study was to characterize the urinary proteome profile of healthy newborn calves. We applied 2-D gel electrophoresis combined with MALDI-TOF-TOF-MS/MS and 1-D gel electrophoresis coupled with LC-MS-MS analysis to separate and identify proteins present in the urine of newborn calves. Urinary proteomic analysis allowed us to detect proteins which are characteristic of embryonic and neonatal development and proteins involved in the cardiovascular and digestive system, and bone and kidney development. Moreover, we identified proteins that can be used to assess normal glomerular and tubular physiology. In calves, one of the main causes of mortality in the neonatal period is diarrhea, affecting fluid and electrolyte balance. We also identified proteins involved in the renal transport of water and electrolytes, which can be useful in the early diagnosis and prevention of these type of disorders.

**Abstract:**

Urine is a biological diagnostic material suitable not only for the analysis of kidney and urinary tract functions but also the function of other tissues and organs. The urine proteome of adult mammals differs from the urine proteome of neonatal ones. The establishment of urinary protein maps of healthy newborn calves is important for diagnosing and monitoring the progression of various diseases. The experiment was carried out on a Polish-Friesian var. of Black-and-White male calves in the sixth day of postnatal life. The two proteomics approaches used for separation and identification of urinary proteins were: 2-DE with MALDI-TOF-TOF-MS/MS and 1-DE with LC-MS/MS. This resulted in the identification of 692 urinary proteins. The majority of them were classified as extracellular proteins (40.32%), as well as proteins involved in regulation of major cellular processes (31.07%). We have observed the presence of unique proteins associated with embryonic (ameloblastin, alpha-fetoprotein, Delta-like protein, embryo-specific fibronectin 1 transcript variant, Indian hedgehog homolog) and kidney development (angiotensin-converting enzyme, angiotensinogen, aquaporin-1, calbindin, glypican 3, nidogen 1, pro-cathepsin H). Additionally, proteins involved in the renal regulation of water and electrolyte balance (angiotensinogen, angiotensin-converting enzyme, aquaporin-1, ezrin, uromodulin) were detected. Presented in the current study 1-D and 2-D urinary proteomic maps are the basis for the identification and detection of prognostic biomarkers important for defining a calf’s health status.

## 1. Introduction

Urine is a body fluid that can be obtained noninvasively in large quantities. Quantitative and qualitative urine protein analyses are commonly used to assess renal function. Moreover, urinary proteins might be a source of biomarkers that may reflect the entire spectrum of diseases such as, chronic kidney disease, obstructive nephropathy, renal cell carcinoma, bladder cancer as well as prostate cancer [1]. Recent studies also suggest that urine may be useful for diagnosing other systemic diseases such as coronary artery disease [2] or gastrointestinal diseases [3]. 

So far, more than 2300 proteins have been identified in human urine [4] and more than 1550 proteins in the urine of Karan Fries cows [5].

A comprehensive characterization of urinary proteome profiles of domestic animals has the potential to identify biomarkers that can be used in veterinary diagnosis. Proteomic urine analysis in domestic cats with chronic kidney disease [6] and in dogs with kidney injuries obtained during babesiosis [7] have allowed for the selection of proteins that could be biomarkers of these diseases. Proteomic research in farm animals focuses mainly on the finding of biomarkers associated with production. For example, analysis of urine proteomic profiles of pregnant cows allowed scientists to isolate a pregnancy-specific protein (bovine pregnancy-associated protein–bPAP) [8]. Many researchers use animal models to elucidate the mechanisms responsible for physiological and pathophysiological processes that occur in humans. For instance, a study of Palviainen et al. [9] provided novel urinary acute kidney injury (AKI) biomarkers in sheep, which may find application in early diagnosis of AKI in humans.

Urinary protein composition highly depends on the physiological, nutritional-related and age-related conditions. Alhaider et al. [10] observed a few differences between the urinary proteomes of lactating, pregnant and virgin camels. According to the authors the hormonal status is not a major factor influencing protein excretion in camel urine. Moreover, analysis of the camel urinary proteome allowed for the identification of proteins involved in various stress and immune response processes, as well as those displaying antimicrobial activities [10]. Our previous study showed that excessive amounts of lactose in the diet of two-week-old calves resulted in altered urinary protein profile [11].

In the available literature there are also studies aimed at analyzing the differences in the urinary proteome profiles between neonate and adult animals. Lee et al. [12] identified two primary groups of proteins differentiating the neonatal and adult rat urine samples. Urinary proteins involved in cellular adhesion, structure, proliferation and differentiation were identified during the early postnatal life and were not present in adult animals. Moreover, the authors showed the presence of proteins associated with male sexual maturation (prostatic- and seminal vesicle-secreted proteins) only in the urine samples collected from the adult rats.

A rapid differentiation and development of organs and systems initiated in the embryonic phase continues throughout the postnatal period. It seems that the morphological and functional changes observed after birth have a significant influence on urine protein composition. Therefore, assessment of the complete urinary proteome in calves may allow scientists to identify proteins that only occur in the neonatal period, including those that reflect normal kidney physiology and other vital organs. 

High mortality is observed during the first months of a calf’s life. Diarrhea, and subsequent water and electrolyte imbalance, are some of the main reasons for neonatal calf mortality [13]. Therefore, the establishment of urine protein maps of healthy newborn calves is important for diagnosing and monitoring the progression of various diseases, including water and electrolyte imbalance.

The aim of this study was to characterize the urinary proteome profile of healthy newborn calves. We applied 2-D gel electrophoresis combined with MALDI-TOF-TOF-MS/MS and 1-D gel electrophoresis coupled with LC-MS-MS analysis to separate and identify proteins present in the urine of newborn calves. 

## 2. Material and Methods

### 2.1. Animals and Diets

The experiment was carried out on 8 Polish-Friesian var. Black-and-White male calves in the sixth day of postnatal life. The animals were under veterinary care and supervision and that they did not show any clinical symptoms of diseases. During the first three days of life, calves were fed with colostrum and then from the 4th day with milk replacer (see Dratwa-Chałupnik et al. [11] for details). Each day the animals were weighed, because the amount of milk replacer was given per calf body weight. Experimental calves came from the same farm, they were of similar body weight, which averaged 38 kg and lived under uniform controlled environmental conditions. The use and handling of animals for this experiment was approved by the Local Commission of Ethics for the Care and Use of Laboratory Animals (Resolution No. 3/2010).

### 2.2. Isolation of Urine Proteins

Urine samples were collected directly into the collection bags attached to the loins three hours before the evening feeding and after five milk replacer feedings. Immediately after draw, urine was centrifuged at 3000 rpm × 10 min at 4 °C to remove cellular debris and the resulting supernatants were frozen at −80 °C until further analysis. The urine was concentrated using Amicon Ultra-15 centrifugal filter devices of 3 kDa cut-off (Merck Millipore, Germany) at 5000 rpm and at 4 °C. Subsequently 100 µL of the final concentrate was precipitated using the ReadyPrep 2-D cleanup kit (BioRad) according to the manufacturer’s protocol.

### 2.3. Sample Preparation and Selection for ProteomicsAnalysis

The urine protein pellet was resuspended in 250 µL of a lysis buffer (7 M urea, 2 M thiourea, 2% Chaps, 0.4% DTT, 0.5% Pharmalyte 3–10, 0.5% IPG 6–11 buffer) and shaken vigorously overnight at room temperature. In order to standardize urine samples, the protein concentration in the samples was determined using the Bradford method (1976), which was adapted for use with a lysis buffer.

All collected urine samples (8) were analyzed by 2-DE and compared visually between each other. Two, that showed patterns most representative for this experiment were selected for further proteomics analysis. Selected protein spots were cut out and analyzed by MALDI-TOF-TOF to identify proteins. The same samples were further used for GEL LC-MS/MS (separated using 1-DE and identified and quantified using LC-MS-MS).

### 2.4. 2-Dimensional Gel Electrophoresis (2-DE)

The first dimension was performed on 18 cm IPG 4–7 strips. Each gel was loaded with 240 µg of proteins. Isoelectrofocusing (IEF) was performed on Multiphor II at 20 °C using a voltage/time profile linearly increasing from 0 to 600 V for 2 h and 15 min, from 600 to 3500 V for 8 h and 3500 V for 9 h and 15 min. Total-volt-hours(Vh) of 2-DE were 50,000. After IEF, the strips were equilibrated for 15 min in equilibration buffer (6 M urea, 2% SDS, 30% glycerol, 50 mM Tris-HCl pH 8.8, 1% DTT). The second dimension was performed using a Protean II XL system (Bio-Rad Laboratories) and laboratory-made 12.50% (w/v) acrylamide gels (acrylamide: *N*,*N*′-ethylene-bis-acrylamide ratio 200:1). The gels were run overnight at 20 °C at a constant current setting (6 mA/gel for 2 h and 9 mA/gel for 10–12 h). To follow the progress of the electrophoresis process we have used Bromophenol blue added to the 1st dimension gel loading solution. The electrophoresis process was terminated when the blue dye was 1 mm from the bottom edge of the 2nd dimension gel glass plate. The running buffer (0.67% Tris-Base, 1.44% Glycine, 0.1% SDS) was recirculated at a flow rate of 4 l/min. After the second-dimension separation, gels were fixed in 45% methanol, 7.5% acetic acid for 1 h and stained in SyproRuby stain for 3 h. After staining, the gels were washed in 15% ethanol, 7.5% acetic acid for 45 min and in water for 10 min. Gel images were captured using the BioSpectrum^®^ 2D Imaging System (UVP, Upland, CA, USA) with an excitation wavelength at 302 nm and a 455–495 nm emission filter.

### 2.5. Protein Spot Identification by Mass Spectrometry

Spots of interest were manually excised from the gels and washed with deionized water followed by two washes with 100% acetonitrile for 15 min and 2 min, respectively. The gel plugs were dehydrated in a vacuum centrifuge and rehydrated with a solution of 2% trypsin in 50 mM NH_4_HCO_3_, at 4 °C. After 20 min, the excess of trypsin solution was removed and 30 µL of 50 mM NH_4_HCO_3_ was added and digestion proceeded overnight at 37 °C, followed by a storage at −20 °C until use.

### 2.6. MALDI-TOF-TOF-MS/MS

Desalting of peptides was performed on custom-made reverse-phase microcolumns, prepared with R2 resin (Perseptive Biosystems Inc., Framingham, MA, USA) as described previously [14]. Peptide solution, obtained from the digestion of each spot, was loaded onto the microcolumn, and washed with 10 µL of 1% trifluoroacetic acid [15]. Bound peptides were eluted with 0.8 µL of matrix solution (5 µg/µL of alpha-cyano-4-hydrocynnamic acid in 70% acetonitrile and 0.1% TFA) directly onto the matrix-assisted laser desorption ionization (MALDI) target plate. Peptide mass spectra were acquired in a positive reflector mode on a 4800 Plus MALDI TOF/TOF™ Analyzer (Applied Biosystems, Foster City, CA, USA) using an acceleration voltage of 20 kV. Each spectrum was obtained with a total of 800 laser shots and was externally calibrated using peptides derived by tryptic digestion of ß-lactoglobulin. Tandem mass spectra were acquired using the same instrument in MS/MS positive mode. Peak lists were generated from the raw data output by Data Explorer (Applied Biosystems, Foster City, CA, USA). MS and MS/MS peak lists were combined into search files and used to search SwissProt 57.7 (497.293 sequences; 175.274.722 residues) and MSDB 20.060.831 (3.239.079 sequences; 1.079.594.700 residues) or NCBInr 20090919 (9.733.153 sequences; 3.324.824.611 residues) using the Mascot search engine (Matrix Science Ltd., London, UK). Search parameters were as follows: Database, SwissProt or MSDB or NCBI; Taxonomy, Bos taurus; Enzyme, trypsin; Allow up to 1 missed cleavage; fixed modifications, none; variable modifications, methionine oxidation; peptide mass tolerance, 70 ppm; and fragment mass tolerance, 0.6 Da.

### 2.7. GEL LC-MS/MS

Samples (16.67 µg proteins per lane) were separated on a Bolt 4–12% Bis-Tris Plus gel using Bolt MES SDS Running Buffer (Life Technologies) following the manufacturer’s instructions. Sypro Ruby Protein gel stain (Life Technologies) was used for the detection of proteins in gel. Protein bands were manually excised from the gels and subjected to proteolytic digestion, as described above. 

Peptide mixtures after digestion were analyzed by reverse phase nanoflow liquid chromatography (EASY-nanoLC, Thermo Fisher Scientific, Waltham, MA, USA) coupled via a nano-electrospray ion source to an LTQ Orbitrap XL mass spectrometer (Thermo Fisher Scientific, USA). Briefly, samples were resuspended in 5 µL solvent A (0.1% FA) and loaded onto a precolumn using intelligent flow control at 260 bar. The precolumn consisted of a 2–3 cm × 100 µm inner diameter fused silica capillary packed in-house with Reprosil Pur C18 AQ, 5 µm (Dr.Maisch, Ammerbuch-Entringen, Germany). Peptides were subsequently separated on an analytical column (15–16 cm × 75 µm inner diameter fused silica capillary with a laser pulled emitter packed in-house with Reprosil Pur C18 AQ, 3 µm (Dr.Maisch, Ammerbuch-Entringen, Germany) in a linear gradient of 0–34% solvent B (95% ACN 0.1% FA) over 30 min at a flow rate of 250 nl/min. Mass spectra were acquired in positive ion mode using data-dependent acquisition. Survey scans in the m/z range 300–1650 were acquired in the Orbitrap at a resolution of 30,000. The top 5 most intense precursor ions per survey scan were selected for fragmentation using collision induced dissociation (CID). A normalized collision energy of 35 was used and fragment ion spectra were acquired in the LTQ. Mass spectra were converted into peak lists by ProteomeDiscoverer software version 1.4 (Thermo Fisher Scientific, Waltham, MA, USA). The abundance of protein was quantified based on the area of the 3 most intense peptides and represented as a relative in sample quantitation of all proteins identified in the experiment. The abundance of each protein was quantified based on the area of the 3 most intense peptides and represented as a relative in sample quantitation of all proteins identified in the experiment as described before in [16,17,18]. This quantification method is part of the Proteome Discover software and was used to identify the most abundant proteins in the calf’s urine.

### 2.8. Functional Annotation and Localization of Identified Proteins 

UniProt [19] was used to infer the molecular functions of the identified proteins. The subcellular localization of proteins was analyzed using Euk-mPLoc 2.0 software [20].

## 3. Results

**Figure 1:**
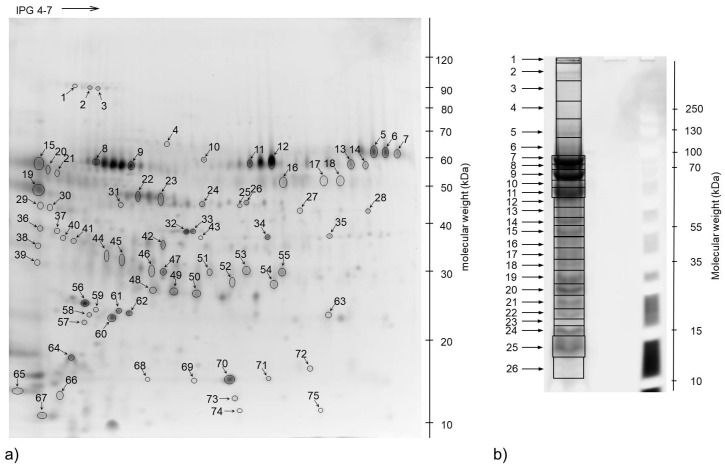
Representative 1-D and 2-D gels: (**a**) Two-dimensional map of calves’ urinary proteome. 2-D gel presents SyproRuby stained urine protein pattern (240 µg of proteins, 4–7 NL 18 cm IPG Strips, 12.5% poliacrylamide gel). Spot IDs correspond to the mass spectrometry identifications presented in Table S1. (**b**) One dimensional gel electrophoresis of proteins isolated from calves’ urine. Proteins were loaded at 16.67 μg per lane and proteins were detected with SyproRuby stain after electrophoresis.

We used two proteomics approaches to the profile urine proteome obtained from eight six-day-old calves. Urinary proteins were separated using either 2D gel electrophoresis and identified by MALDI-TOF-TOF (Figure 1a) or 1D gel electrophoresis followed by ESI-LC-MS-MS (Figure 1b), only the two most representative samples were used for this in-depth analysis. Out of 75 protein spots that were excised from the 2D gels, 32 were successfully identified. Two spots contained more than one protein and 17 proteins were present in more than one spot. 

**Figure 2:**
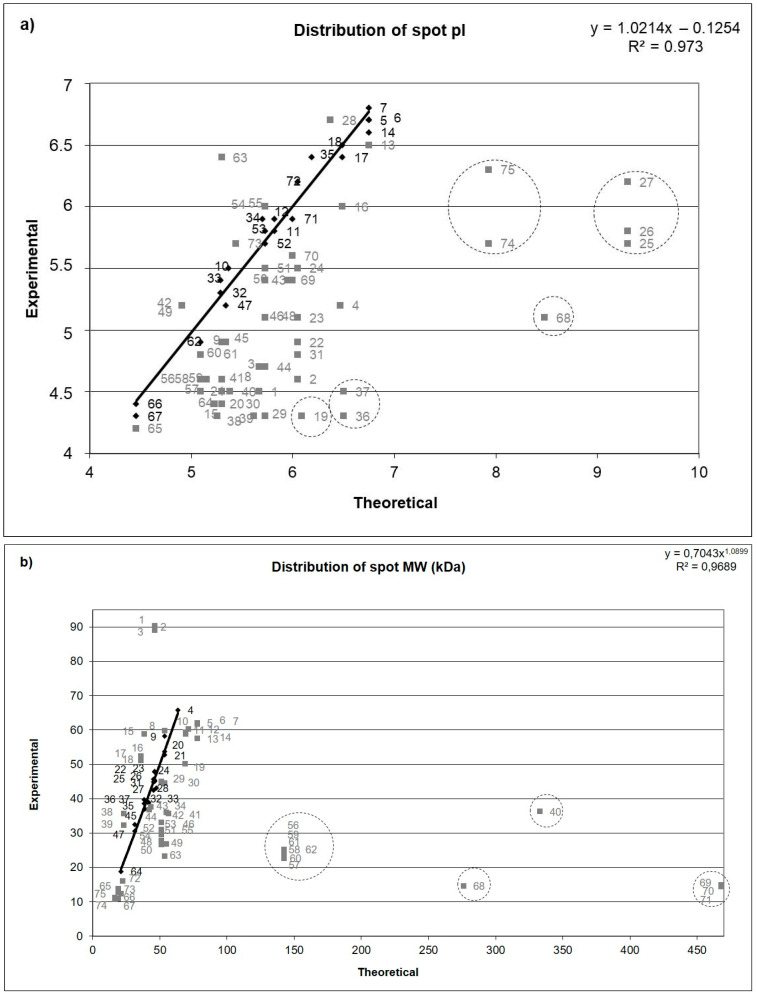
The graphs illustrate differences between proteins’ spot position on the gel and the theoretically calculated protein pI (**a**) and molecular weight (**b**). Spots used for the curve preparation and other plotted spots are marked with black rhombs and grey squares, respectively. Spots found as outliers are circled with dashed line. Theoretically and calculated protein of pI and molecular weight, differences between calculated and theoretical MW and pI, potential modifications are presented in Table S1.

Interestingly, a large discrepancy between the spot position on the gel and the theoretically calculated protein isoelectric point–pI (five proteins) and molecular mass (four proteins) were observed in case of selected proteins (Figure 2a,b).

The list of all identified proteins with detailed mass spectrometry results including the accession number, mascot score, sequence coverage, the number of matched and sequenced peptides, theoretical pI and molecular weight is presented in Supplementary Table S1. 

In order to expand the inventory of identified urinary proteins, we have carried out protein mapping experiment using 1D GEL LC-MS/MS approach. The list of the identified proteins and details of mass spectrometry results are presented in Supplementary Table S2. 

Those two proteomic methods represent different methodological approaches and deliver complementary results due to different physicochemical properties of proteins [21,22]. The 2DE allows for separation and detection of mostly soluble proteins with a molecular weight between 150 and 10 kDa. The Gel LC-MS/MS approach promotes the identification of larger proteins with several trans membrane spans. Application of the two methods had been shown to increase the coverage of proteomes especially when working with samples from organisms with un-sequenced or poorly annotated genomes, which is the case for Bos Taurus. The using of two different proteomic techniques in the presented study increased the number of detected proteins in calf urine. In total, two proteomics approaches allowed us to identify 692 proteins in the urine of six-day-old calves. Ten proteins were identified solely with the aid of 2D gel combined with MALDI-TOF-TOF, 22 were identified by both approaches, and the 1D GEL LC-MS/MS approach allowed us to identify an additional 670 proteins (Supplementary Figure S1). 

### 3.1. General Molecular Function and Subcellular Localization

In order to understand the characteristics of the urinary proteome of six-day-old calves, we analyzed a general molecular function and subcellular localization of all of the identified proteins. The detailed data concerning protein function and localization are presented in Supplementary Table S3.

**Figure 3:**
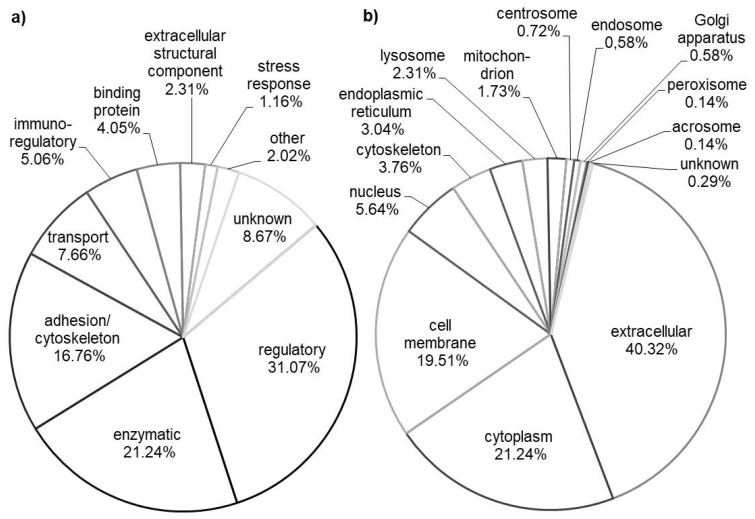
Biological function (**a**) and subcellular localization (**b**) of calf urinary proteome components.

The majority of identified proteins were categorized as extracellular (40.32%), cytoplasmic (21%) and cell membrane proteins (19%), Figure 3b.

The largest group of proteins was involved in various regulatory processes (31.07%), Figure 3a. Enzymes (21%) and proteins involved in cell adhesion and organization of the cytoskeleton (16%) were the next two largest groups.

### 3.2. Tissue Origin and Organs Development

Most of the proteins found in the urine originated from the blood plasma and kidneys. Therefore, in order to understand the characteristics of urinary proteome of newborn calves we have described their tissue origin and relation to organ development (see Table 1 and Table 2). 

The main tissue origin groups were blood plasma proteins detected in normal urine, proteins detected in bovine blood plasma/serum, proteins obtained in the blood plasma of seven-day-old calves, proteins detected in the bovine kidney and proteins present in the urine only during neonatal period [23,24,25].

The proteins related to organs’ (systems) development were mainly represented by the cardiovascular system development group (actin, alpha skeletal muscle; angiopoietins; angiotensinogen) and kidney system development group (angiotensin-converting enzyme, angiotensinogen, aquaporin-1, calbindin).

### 3.3. The Most Abundant Proteins

LC-MS/MS analysis combined with the top three label free quantitation approach implemented in the Proteome Discoverer software (Thermo Scientific) allowed us to estimate the within urine sample relative abundance of proteins. We found that cathelicidins were among the most abundant proteins. Protein bands 24, 25 and 26 contained those proteins (Supplementary Table S4).

Other highly abundant proteins were acute phase proteins (Serpin A3-1, Alpha-2-HS-glycoprotein, Alpha-1-acid glycoprotein, Prothrombin), ion transport and ion binding proteins (serotransferrin, lactoferrin, serum albumin, carboxypeptidase B2), proteins known as indicators of kidney function (uromodulin and clusterin), proteins participating in the defense response to bacteria (cathelicidins, lysozyme C, non-stomach isozyme) as well as cytoskeletal and adhesion proteins (keratin, dermatopontin). Serpin A3-1, an acute phase protein, was the most abundant protein in the band five. Clusterin was found to be the most abundant protein in the band 15.

## 4. Discussion

The most intense functional changes to extra-uterine life occur during the first week of life. During that time of postnatal adaptation systems such as the digestive, excretory, respiratory and circulatory systems have to rapidly adjust to the new environment. Effectiveness and completeness of those changes affect the health and survival chances of newborn calves dramatically, and, therefore, this period of life is very important from a veterinary and nutritional perspective.

Our previous research focused on studying calf kidney function during the first week of life [26,27,28,29]. It has been shown that during this period dynamic changes occur in the kidney function and in its hormones regulating profile.

Considering the fact that many factors like age, nutrition and physiological condition may potentially influence the protein profile of body fluids, the current study was performed on six-day-old calves, which after birth were fed mother’s milk, and subsequently milk replacer three days before the samples were collected, to unify the nutritional factor. Colostrum intake by calves is necessary to acquire an immune response to pathogens. The obtained protein profile of calf urine can be used for comparison with the protein profile of older animals which are fed with a milk replacer.

Previous proteomic studies of calf blood plasma [30] have shown a significant reduction in apoA-IV expression on the first day, when changing from milk feeding to milk replacer. During the next days of life, the expression of this protein increased. Moreover, during feeding with milk replacer a lower expression of apo-I was observed in comparison to calves fed with milk. No qualitative changes in protein composition were observed. It could be expected that the milk replacer does not change the protein composition of urine.

### 4.1. Proteomics Characterization of Newborn Calf Urine

After blood plasma, urine is the second most frequently analyzed body fluid in veterinary diagnosis. It is essential to establish the basal protein composition of urine of healthy animals at different stages of normal development to efficiently identify disease markers in urine. Therefore, this study attempts to characterize the urinary proteome of six-day-old calves.

Filtered and excreted proteins in the urinary tract are present in the final urine. It is estimated that 70% of urinary proteins derive from the kidney and urinary tract, whereas the remaining 30% represent filtered plasma proteins [31]. In newborns, due to leakage of the filtration barrier and the limited resorption process, proteins that are absent in the urine of healthy adults could be expected. In the present study we applied two different proteomic strategies, i.e., 2-DE combined with MALDI-TOF-TOF-MS/MS and 1-DE associated with LC-MS/MS to globally characterize the urinary proteome of a healthy neonatal calf.

The differences between the theoretical MW and the MW observed in the 2-D gels were observed for the most of the identified protein spots. This may potentially suggest the occurrence of posttranslational events modulating those proteins. According to Bouley et al. [32] the differences in protein’s molecular weight can be the result of different types of posttranslational modifications, such as phosphorylation, glycosylation and proteolytic cleavage. Therefore, it seems possible that in the present study also, posttranslational modifications were mainly responsible for the observed discrepancies in the MWs (Supplementary Table S1). For example, spots 69, 70 and 71 were identified as a basement membrane-specific heparan sulfate proteoglycan core protein. The position of those spots suggests that this protein is cleaved. Previous work of Pieper et al. [33] has shown that such a modification of the sequence of that protein is possible.

### 4.2. Comparison to the Human and Bovine Urinary Proteome and Bovine Kidney Proteome

In the available literature there are no proteomic studies aimed at analyzing protein composition of urine from calves. In mature cattle, urinary profile has been determined in a single study in Karan Fries cows [5]. At the same time, there exist several comprehensive human urinary proteomic studies [4,34]. Therefore, we have decided to compare the composition and characteristics of a calf’s urinary proteome with the human and cow. The identified proteins in calf urine were compared with the urine proteins of adult cows of a different breed and the proteins of human urine manually on the basis of their naming and accession number. Additionally, in order to estimate which urinary proteins derive from the kidney, we have compared it with the bovine kidney proteome [24].

**Figure 4:**
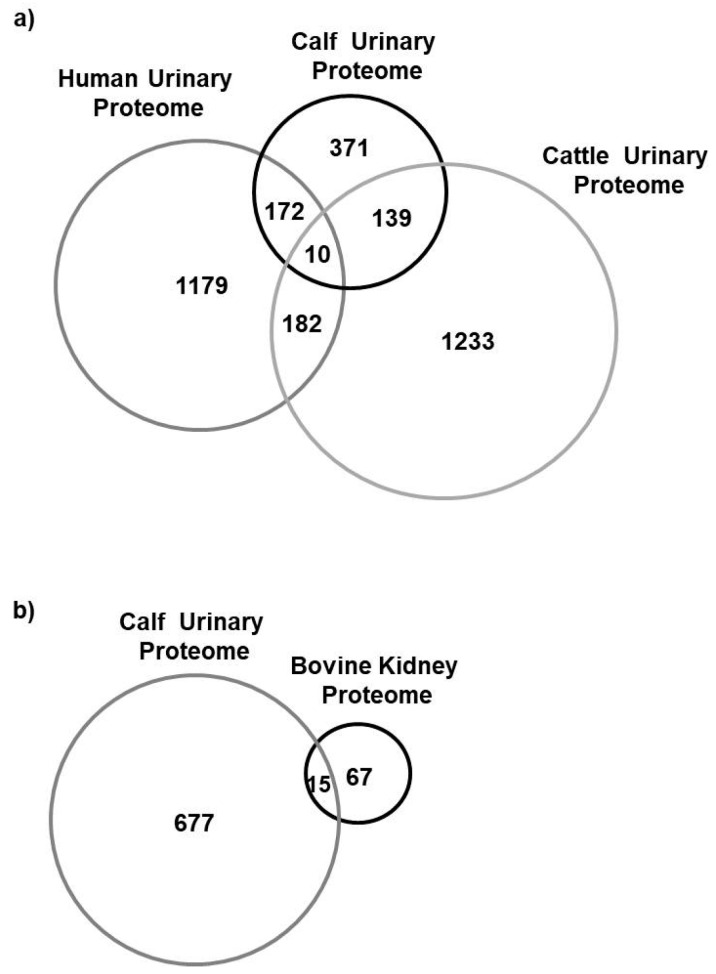
Comparison of human, bovine and calf urinary proteome (**a**) [5,34], comparison of calves’ urinary proteome with kidney proteome (**b**) [24].

We identified 692 calves’ urinary proteins that were also present in human urine [34], in bovine urine [5] and those identified in the bovine kidney [24]. Additionally, we showed that 182 out of 1543 human urine proteins [34], 149 out of 1550 cows’ urine proteins and 15 out of 82 of the bovine kidney proteins [24] were also detected in calves’ urine (Figure 4). The lower amount of identified proteins in calf urine compared to cattle may result from the lower possibility of urine concentration compared to adult individuals [27]. In addition, urine is a body fluid poor in protein and rich in salts. Therefore, urine samples should be concentrated and desalinated before proteomic analysis. A low amount of protein in samples may make it impossible to identify it.

A small amount of common proteins for three different types of urine samples may result from various preparation of samples for proteomic analyses and the proteomic techniques used [21,22]. In addition, differences in the protein composition between animal breeds and species are observed. For example, Pasha et al., [35] using quantitative mass spectrometry, has demonstrated differences in the protein composition of saliva between breeds of dogs. These differences were smaller compared to human saliva.

### 4.3. The Largest Group of Proteins Involved in Various Regulatory Processes

In this group, we found the presence of proteins displaying a wide spectrum of biological activities involved in the regulatory processes within cellular organelles, whole cells, tissues and organs. Among them were proteins involved in the regulation of cell proliferation (Inhibin alpha chain), growth and shape (cochlin, HPN protein), differentiation (delta-like protein), migration (migration and invasion enhancer (1) as well as death and the apoptotic process (clusterin). Another group includes proteins controlling anabolic and catabolic processes and those engaged in the metabolic regulation of lipids, proteins and carbohydrates (epididymal secretory protein E1, glypican 3, Insulin-like growth factor (2). We also identified proteins involved in the regulation of gene expression, transcription, translation and protein folding (major prion protein). As well as those engaged in the regulation of receptor activity, the receptor signaling pathway and signal transduction (epidermal growth factor receptor, tyrosine-protein kinase receptor). Another group comprises proteins involved in the control of proteolysis (hepatocyte growth factor-like protein), enzyme activity (serpins) and those implicated in the regulation of inflammatory responses (pro-cathepsin H, uteroglobin) and phagocytosis (alpha-2-HS-glycoprotein). Additionally, we detected proteins involved in the regulation of blood coagulation (fibrinogen, prothrombin, von Willebrand factor), the production of hormones (angiotensin-converting enzyme, angiotensinogen) and those responsible for the regulation of different tissues, organs and systems’ functions: brown fat (Leucine-rich alpha-2-glycoprotein 1) kidney (uromodulin), heart and vascular system (myoglobin, angiotensinogen), bone and skeletal system (osteomodulin), digestive system (gastrin-releasing peptide), nervous system (midkine), reproductive system (spermadhesin-1).

### 4.4. Blood Plasma-Derived Proteins in Urine

The glomerulus filters the blood that enters the kidney and therefore urine abounds in blood-derived proteins. Moreover, it should be also emphasized that both in human and animal newborns neonatal proteinuria is observed that refers to higher permeability of the filtration barrier and thus results in increased urinary protein excretion [36]. This is consistent with our present study as we identified the blood plasma-specific proteins in the calves’ urine (Table 1). We found that 26 different proteins identified in the urine of calves were also present in the bovine blood plasma/serum [23,24,25] (Table 1). In total, 15 urinary proteins revealed in our study were also detected in the blood plasma of the seven-day-old calves [23] (Table 1). Of the urine proteins that could indicate the permeability of the filter barrier, von Willebrand factor with a molecular weight of 307.5 kDa and aminopeptidase N with a molecular weight of 109.3 kDa could be mentioned.

### 4.5. Kidney-Specific Urinary Proteins

The majority of proteins present in urine derive from the kidney. Some of the urinary proteins detected in our study were also identified in the bovine kidney by Talamo et al. [24] (Figure 4b).

Furthermore, in the urine of six-day-old calves proteins involved in early development of the kidneys were shown. Our data demonstrated the presence of a Delta-like protein, a specific ligand of the Notch receptor. Notch signaling is important in cell fate determination and differentiation during development. The interaction of the Notch receptor with the Delta-like protein participates in nephrogenesis [37]. The presence of this ligand was shown in rat urine during early postnatal maturation but not in 30-day-old rats [12].

Additionally, our current study also revealed the appearance of other urinary proteins that are involved in cell adhesion, structure, proliferation and differentiation. Those proteins were also detected by Lee et al. [12] in rat urine during the neonatal period (Table 1).

The presence of urinary proteins involved in renal development in calves indicates that extensive morphological changes occur in this organ. This reflects a dynamic change of kidney function that take place with calves’ age [26].

### 4.6. Proteins Related to the Mammalian Embryonic Development

In our study we also confirmed that the urinary proteome contains proteins related to the mammalian embryonic development, including ameloblastin, a Delta-like protein, embryo-specific fibronectin 1 transcript variant and Indian hedgehog homolog (Table 2). Those proteins were neither detected in adult human nor mature cattle urine [5,34].

Ameloblastin is a protein involved in a biomineral tissue development, i.e., osteogenesis [38].

Embryo-specific fibronectin 1 transcript variant is one of fibronectin 1 isoforms. According to Goossens et al. [39], the presence of this protein in the bovine morulae and blastocysts may indicate that it is involved in the process of compaction and blastocyst formation.

Hedgehog (Hh) family proteins are secreted molecules that have a role in cell growth and differentiation during embryonic development [40]. In mammals, there are three Hh proteins: Sonic Hh, Indian Hh and Desert Hh. Hedgehog signaling is most active during embryogenesis. A study performed on healthy mice showed that Hedgehog signaling is inhibited in the adult kidney [40].

However, further studies are required to define the exact age of calves when no proteins related to embryonal development will be detected in the urine.

In the urine, we also detected the presence of proteins that can be potentially used as biomarkers of proximal tubular (retinol-binding protein) and distal tubular (uromodulin) function evaluation [41] (Table 2). It should be pointed out that a retinol-binding protein was not detected in the urine of adult cattle [5].

During the assessment of the efficiency of the newborn calf kidney’s functions, it should be considered that they show many morphological and functional distinctness, in comparison to the mature individuals. The results of previous studies indicate that the renal blood flow, glomerular filtration rate as well as tubular resorption and excretion are lower in newborn calves [26].

### 4.7. Proteins Involved in Kidney Regulation of Water and Electrolyte Balance

Analysis of calves’ urinary proteome revealed the presence of proteins involved in the kidney regulation of water and electrolyte balance, including angiotensinogen, uromodulin and three proteins which were absent in adult cow urine [5]: angiotensin-converting enzyme, aquaporin-1 and erzin (Table 2). In turn, aqaporin-10 [5] detected in the urine of mature cattle was not present in the urine of newborn calves.

Angiotensinogen is a component of the renin-angiotensin system (RAS). Plasma angiotensinogen is primarily produced and secreted by the liver. The kidney also produces angiotensinogen in the proximal tubular cells, and it is directly released into the tubule [42]. The research on rats showed that urinary angiotensinogen is secreted by the proximal tubules but it does not derive from the blood plasma [43]. Renin is primarily released from the juxtaglomerular cells of the glomerular afferent arterioles and cleaves the circulating angiotensinogen to the form of decapeptide, angiotensin (Ang) I [44]. This decapeptide is rapidly converted by the angiotensin-converting enzyme (ACE) to the octapeptide, angiotensin (Ang) II [44]. Ang II stimulates synthesis and release of aldosterone, which stimulates sodium reabsorption, primarily through the mineralocorticoid receptors in the collecting tubules. In the renal collecting duct, urinary angiotensinogen can be converted to Ang I and Ang II by the tubular renin and the local ACE [42]. Ang II directly stimulates sodium channels in the kidney collecting ducts [45]. Angiotensinogen is considered as a potential urinary biomarker that identifies humans from group of risk of chronic kidney disease (CKD) [46].

In normal urine there is a relatively high concentration of uromodulin (or Tamm-Horsfall protein). Its biological function is still not fully understood. Uromodulin is most likely involved in the regulation of water and electrolyte balance and kidney innate immunity. Recent studies suggest that the level of uromodulin in the urine could be used as a biomarker of the development of chronic kidney diseases [47].

AQP1 plays an important role in renal water retention. In bovine kidney, AQP1 was detected in the apical and basal plasma membranes in the epithelial cells of the proximal tubule and the thin descending limb of Henle’s loop [48]. The participation in cell migration including the response of the renal proximal tubule cells to injury is also ascribed to the AQP1 [49].

Ezrin binds to actin and the Na+/H+ exchanger regulatory factor (NHERF) and forms a multiprotein complex with Na+/H+ exchanger type 3 (NHE3). Formation of this complex facilitates NHE3 phosphorylation and inhibits N+/H+ exchange, resulting in the inhibition of NaCl and NaHCO_3_ reabsorption in the proximal tubules [50]. Study on rats after acute sodium loading indicated that this protein plays important roles in the transport of sodium in kidney tubules [31].

Proteins involved in the kidney regulation of water and electrolyte balance could be a diagnostic and prognostic support in prophylaxis and (or) prevention in animals husbandry and veterinary medicine.

### 4.8. Proteins Involved in Extrauterine Development and Proteins Characteristic of Various Systems (Organs)

During the neonatal period, the morphological and functional development of many organs and systems important for a growing organism occurs. This stays in accordance with the results of our study as we detected many urinary proteins involved in the development of the cardiovascular system, bone, kidney and the digestive system (Table 2).

Calves’ urine contained proteins that are typical for tissues other than kidney; that indicates that urine could also be used to diagnose the activities of nervous, cardiovascular and skeletal systems (Table 2).

The presence of those proteins in urine could be useful in health status monitoring of growing animals.

Interestingly, a relatively high concentration of cathelicidins in calves’ urine was shown (Table S4). Cathelicidin is expressed in circulating neutrophils and myeloid bone marrow cells, in epithelial cells of the skin, the gastrointestinal tract and urinary tract, as well as in the lungs [51]. Cathelicidins stimulate both innate and adaptive immune responses. A suggested function of this protein is its participation in immune processes during a urinary tract infection; direct antimicrobial defense of epithelium, recruitment of immune system cells and killing of bacteria by neutrophils [51].

Infection of the urinary tract induces the rapid secretion of cathelicidin by the epithelial cells [51]. Previous studies on humans demonstrated that cathelicidin is continuously synthesized by the tubular epithelium despite the absence of microbes [51]. Chromek et al. [51] indicated that this peptide provides the first line of defense against attaching the bacteria. These authors suggest that the antimicrobial peptides of the cathelicidins family are important in bacterial clearance of the urinary tract.

The concentration of cathelicidins in the urine of healthy cattle is low [52]. We hypothesize that substantial amount of cathelicidins found in newborn calves’ urine may play a defensive and protective role of the urinary tract against bacterial infections.

### 4.9. The Most Abundant Proteins

Apart from cathelicidins, other abundant proteins such as serpin A3-1 and clusterin were present in calf urine (Supplementary Table S4). Serpin A3-1 also named as α1-antichymotrypsin (ACT) is a member of the serine proteinase inhibitors. ACT was found in bovine physiological fluids and various tissues, including the blood plasma, urine, heart, liver, lung, kidney, testis, spleen, thymus, cerebellum and muscle [53]. Its main physiological role is to inhibit neutrophil elastase and it contributes to the innate immune system as an anti-inflammatory protein [53]. Its expression is increased during inflammation. Concentration of this protein is higher in the urine of adult humans than in infants [54].

Clusterin is synthesized after several types of tubular injury. It may help to differentiate between tubular and glomerular forms of proteinuria [55]. Our previous studies on goats [36] and calves (data not published) have shown the presence of neonatal proteinuria in those animals. It seems that increased neonatal urinary protein excretion results from immaturity of the filtration barrier as well as the reabsorption systems in the tubules. Interestingly, Saeidi et al. [56] observed a decrease in urinary clusterin concentration with postnatal age. Therefore, we suggest clusterin as a potential urine biomarker of maturity and functionality of the filtration barrier and the tubular reabsorption system in neonates of farm animals.

### 4.10. Urinary Proteomics in Veterinary Medicine

In veterinary medicine, the application of proteomics techniques is still limited. Research determining the protein composition of farm animal urine may be the starting point for future research focused on the comparison of the urine proteome the during physiological condition with the urine proteome in specific clinical pathologies.

Calf diarrhea is one of the main causes of high morbidity and mortality and an important cause of economic losses in the livestock industry [57]. Causes of diarrhea include different factors: environmental (e.g., improper animal hygiene), nutritional (e.g., excessive amounts of milk or milk replacer) and infective (*Escherichia coli*, Cryptosporidium, Rotavirus and to a lesser extent Corona virus).

The results of the previous studies [11] indicate that an excessive amount of lactose in a diet of calves caused osmotic diarrhea. Proteomic analysis of the urine showed changes in the protein profile after administration of lactose to the milk replacer.

Studying changes in the calf urine protein profile during pathogen-induced diarrhea can help to determine the pattern of urine proteins characteristic of a calf with diarrhea induced by a specific pathogen. Furthermore, determining the pattern of indicator proteins could help to determine the cause of diarrhea. The determination by using proteomic techniques of indicator proteins for a specific disorder may provide diagnostic and prognostic support for zootechnical and veterinary prevention.

## 5. Conclusions

Urine is an underappreciated biological sample in the context of health monitoring of newborn calves. It can be collected noninvasively and used for diagnostic purposes, which is an attractive option in veterinary examination.

Urinary proteomic analysis allowed us to detect proteins, which are characteristic of embryonic and neonatal development and proteins involved in cardiovascular, digestive system, bone and kidney development. Moreover, we identified proteins that can be used to assess normal glomerular and tubular physiology.

In calves, one of the main causes of mortality in the neonatal period is loss of water and electrolytes by the digestive tract, that contributes to a failure of water–electrolyte balance. We also identified proteins involved in renal transport of water and electrolytes, which can be useful in early diagnosis and prevention of these type of disorders.

## Figures and Tables

**Table 1:** Tissue origin of proteins identified in the urine of newborn calves (detailed list of proteins with their accession numbers is given in Supplementary Table S3). UniProt [19] was used to infer the molecular functions of the identified proteins.

**Blood Plasma Proteins Detected in Normal Urine**	
alpha-1-acid glycoprotein	hemogloglobin alpha and beta chain
alpha-1-antiproteinase	immunoglobulins
alpha-1B-glycoprotein	kininogen-2
aminopeptidase N	leucine-rich alpha-2-glycoprotein precursor
angiotensinogen	retinol-binding protein 4
apolipoproteins	serotransferrin
clusterin	serum albumin
**Proteins Detected in Bovine Blood Plasma/Serum**	
78 kDa glucose-regulated protein	haptoglobin
actin, cytoplasmic 1	immunoglobulin gamma 1 heavy chain constant region
alpha-1-antiproteinase	kininogen-1
alpha-2-HS-glycoprotein	kininogen-2
apolipoprotein A-I	plasminogen
apolipoprotein A-IV	prothrombin
apolipoprotein C-III	retinol-binding protein 4
apolipoprotein E	serotransferrin
clusterin	serpin A3-1
fibrinogen alpha chain	serum albumin
fibrinogen beta chain	transthyretin
fibrinogen-like protein 1	vitamin D-binding protein
gelsolin	
**Proteins Obtained in Blood Plasma of Seven-Day-Old Calves**	
78 kDa glucose-regulated protein	fibrinogen-like protein 1
actin, cytoplasmic 1	gelsolin
alpha-1-antiproteinase	prothrombin
apolipoprotein A-I	serotransferrin
apolipoprotein A-IV	serum albumin
apolipoprotein E	vitamin D-binding protein
clusterin	von Willebrand factor
fibrinogen alpha chain	
**Proteins Detected in Bovine Kidney**	
acyl-protein thioesterase 1	heat shock cognate 71 kDa protein
alcohol dehydrogenase	peroxiredoxin-2
aldose reductase	phosphoglycerate kinase 1
apolipoprotein A-I	serum albumin
calbindin	superoxide dismutase (Cu-Zn)
carbonic anhydrase 2	triosephosphate isomerase
glutathione S-transferase P	vimentin
glyceraldehyde-3-phosphate dehydrogenase	
**Proteins Detected Only During Neonatal Period**	
ameloblastin	gelsolin
Delta-like protein	lysyl oxidase
embryo-specific fibronectin 1 transcript variant	

**Table 2:** Functional groups of newborn calves’ urinary proteins (particularly information about urinary proteins are shown in Supplementary Table S3). UniProt [19] was used to infer the molecular functions of the identified proteins.

**Mammalian Embryonic Development**	
ameloblastin	embryo-specific fibronectin 1 transcript variant
alpha-fetoprotein	Indian hedgehog homolog
Delta-like protein	
**Assessment of the Renal Tubules Function**	
retinol-binding protein	uromodulin
**Regulation of Water and Electrolyte Balance in Kidneys**	
angiotensinogen	ezrin
angiotensin-converting enzyme	uromodulin
aquaporin-1	
**Cardiovascular System Development**	
actin, alpha skeletal muscle	erythropoietin receptor (Fragment)
actin, cytoplasmic 1	glucose-6-phosphate isomerase
alpha-2-antiplasmin	glypican 3
angiogenin-2	hypothetical LOC506714
angiopoietin-4	junction plakoglobin
angiopoietin-like 3	lactadherin
angiotensinogen	lysosomal alpha-glucosidase
annexin A2	myoglobin
apolipoprotein E	retinol-binding protein 4
aquaporin-1	roundabout homolog 4
ATP synthase subunit beta, mitochondrial	serine peptidase inhibitor
beta-1,4-galactosyltransferase 1	thy-1 cell surface antigen
cadherin-2	tyrosine-protein kinase receptor Tie-1
collagen alpha-1(I) chain	ephrin-A1
connective tissue growth factor	erythropoietin receptor (Fragment)
ephrin-A1	
**Bone Development**	
cartilage oligomeric matrix protein	lactoferrin
cell adhesion molecule 1	latent-transforming growth factor beta-binding protein 2
collagen alpha-1(I) chain	secreted protein acidic and rich in cysteine (SPARC)
discoidin domain receptor family, member 2 (DDR2 protein)	
**Kidney Development**	
angiotensin-converting enzyme	glypican 3
angiotensinogen	nidogen 1
aquaporin-1	pro-cathepsin H
calbindin	
**Digestive System Development**	
adipocyte-specific adhesion molecule (ASAM protein)	hypothetical LOC506714
gastrin-releasing peptide	insulin-like growth factor 2 preproprotein
**Nervous System**	
14-3-3 protein epsilon	NAGLU protein
midkine	
**Cardiovascular System**	
angiogenin-2	glutamyl aminopeptidase
angiopoietin	lactadherin (BP47)
ephrin-a1	lysosomal alpha-glucosidase
**Skeletal System**	
collagen alpha-1(I) chain	secreted phosphoprotein 24
collagen triple helix repeat containing 1	tripeptidyl-peptidase 1
glypican 3	

## Supplementary Materials

The following are available online at https://www.mdpi.com/2076-2615/10/8/1257/s1, Table S1. Summary of protein identification from the urine of 6 days old calves identified using MALDI-TOF-TOF-MS/MS, Table S2. Summary of protein identification from the urine of 6 days old calves identified using LC-MS/MS ORBITRAP mass spectrometer, Table S3. Subcellular localization and biological function of calf urinary proteome components, Table S4. Most abundant proteins respective bands of calves’ urine identified using LC-MS/MS ORBITRAP mass spectrometer, Figure S1. Overlap between the 2D gel and 1D LC-MSMS proteomes.
